# Statins Associated with Better Long-Term Outcomes in Aged Hospitalized Patients with COPD: A Real-World Experience from Pay-for-Performance Program

**DOI:** 10.3390/jpm12020299

**Published:** 2022-02-17

**Authors:** Ying-Yi Chen, Tsai-Chung Li, Chia-Ing Li, Shih-Pin Lin, Pin-Kuei Fu

**Affiliations:** 1 Department of Public Health, China Medical University, Taichung 406040, Taiwan; yingyivicky@gmail.com (Y.-Y.C.); tcli@mail.cmu.edu.tw (T.-C.L.); 2 Department of Healthcare Administration, College of Medical and Health Science, Asia University, Taichung 413305, Taiwan; 3 Department of Medical Research, China Medical University Hospital, Taichung 404332, Taiwan; t6446@mail.cmuh.org.tw; 4 Department of Information Engineering and Computer Science, Feng Chia University, Taichung 407802, Taiwan; yalebin.lin@gmail.com; 5 Department of Critical Care Medicine, Taichung Veterans General Hospital, Taichung 407219, Taiwan; 6 Ph.D. Program in Translational Medicine, National Chung Hsing University, Taichung 402010, Taiwan; 7 College of Human Science and Social Innovation, Hungkuang University, Taichung 433304, Taiwan

**Keywords:** aged, COPD, coronary artery disease, statins, long-term outcomes, mortality, pay-for-performance

## Abstract

Chronic obstructive pulmonary disease (COPD) is the third leading cause of death globally. Previous studies have addressed the impact of comorbidity on short-term mortality in patients with COPD. However, the prevalence of cardiovascular disease (CVD) and the association of statins prescription with mortality for aged COPD patients remains unclear. We enrolled 296 aged, hospitalized patients who were monitored in the pay-for-performance (P-4-P) program of COPD. Factors associated with long-term mortality were identified by Cox regression analysis. The median age of the study cohort was 80 years old, and the prevalence of coronary artery disease (CAD) and statins prescriptions were 16.6% and 31.4%, respectively. The mortality rate of the median 3-year follow-up was 51.4%. Through multivariate analysis, body mass index (BMI), statin prescription, and events of respiratory failure were associated with long-term mortality. A Cox analysis showed that statins prescription was associated with lower mortality (hazard ratio (HR): 0.5, 95% Confident interval, 95% CI: 0.34–0.73, *p* = 0.0004) and subgroup analysis showed that rosuvastatin prescription had protective effect on long-term mortality (HR: 0.44; 95% CI: 0.20–0.97; *p* < 0.05). Statin prescriptions might be associated with better long-term survival in aged COPD patients, especially those who experienced an acute exacerbation of COPD (AECOPD) who require hospitalization.

## 1. Introduction

Chronic obstructive pulmonary disease (COPD), the third leading cause of death globally, is a chronic inflammatory disease of the lower respiratory tract that causes progressive and damaging airflow obstruction [1,2,3,4]. The estimated global prevalence of COPD was approximately 11.7% in 2010 [5], and the mortality of COPD is projected to increase to more than 5.4 million annually by 2060 due to the aging populations increased in our society [6,7]. As COPD progresses, aged patients are usually accompanied by multiple comorbidities and frequently experience an acute exacerbation of chronic obstructive pulmonary disease (AECOPD) that results in lung function deterioration and mortality [8]. Accordingly, the most common cause of hospitalization of COPD is AECOPD, which is a devastating event known as the “stroke of the lungs” that requires treatment with systemic glucocorticoids and antibiotics [9,10,11]. In addition, cardiovascular diseases (CVD) are highly prevalent in COPD patients and are a clinically relevant cause of morbidity and mortality [12].

Factors associated with short-term mortality in hospitalized patients with COPD include old age, progression to respiratory failure requiring mechanical ventilation, illness severity, and comorbidities [12,13,14,15]. For long-term mortality prediction, factors including age, dyspnea, airflow obstruction, and multiple comorbidities were identified by previous studies [16,17,18,19]. Statins have been used as a potential adjunct therapy for patients with CVD and hyperlipidemia; however, the additional effects of statins on long-term outcomes of patients with COPD, especially those hospitalized for AECOPD, are still controversial [20,21]. The aim of this study is to investigate the association between statins use and long-term outcomes of hospitalized patients with COPD in a long-term follow-up cohort from the pay-for-performance (P-4-P) program.

## 2. Materials and Methods

### 2.1. Study Design and Ethical Considerations

The retrospective cohort study was conducted in Taichung Veterans General Hospital (TCVGH), a 1200-bed tertiary referral center in Taiwan, from January 2016 to December 2018. The medical information of enrolled patients was retrieved from the Taichung Veterans General Hospital Clinical Research Database (TCVGH-CRD) and included comorbidities, diabetes status, medication history, pulmonary function test results, COPD severity, and blood glucose measurements. The TCVGH-CRD contains five data sets, each containing data on geriatric conditions, health examinations, health insurance claims, internal clinical tests, and cancer registry information [22]. The database has been used for insurance payouts and for research on COPD and critical illnesses. All methods were performed in accordance with the Declaration of Helsinki and the relevant regulations and guidelines [23]. The study was approved by Institutional Review Board I (IRB number, CE20237A; date of approval, 4 July 2020) of TCVGH, and the need to obtain written informed consent was waived because of the retrospective study design and de-identification of patient data prior to analysis.

### 2.2. Patient Enrollment, Grouping, and Definition

Patients who were diagnosed with COPD and had been enrolled in the P-4-P program for COPD for a regular follow-up, over 65 years old, and were hospitalized for AECOPD were enrolled in this study. The definition of AECOPD was patients with COPD required an emergency department visit lasting more than 24 h or new hospital admission and treatment with systemic glucocorticoids and antibiotics [24,25]. We enrolled aged patients who had a regular HbA1c check-up prior to hospital admission for the purpose of diagnosis and follow-up of diabetes mellitus (DM) and metabolic syndrome. The grouping of COPD was classified according to the Global Initiative for Chronic Obstructive Lung Disease (GOLD) report published in 2022, which was based on an evaluation of symptoms and the risk of exacerbation [26].

### 2.3. Data Collection, Assessment, and Outcome Measures

Data on the following variables were collected: age; sex; BMI; medication history for metabolic syndrome, including oral hypoglycemic medication, insulin, and statins; and the Charlson comorbidity index (CCI). The index day of enrollment was the first episode of hospital admission date, at any time between January 2016 and December 2017. The prevalence of intensive care unit (ICU) admission, the status of respiratory failure requiring mechanical ventilation (MV), and hospital mortality were analyzed. The follow-up time for mortality was from the index day to 31 December 2019. The primary endpoint was to evaluate the association between statins prescription and long-term mortality of aged patients with COPD after discharging from the hospital. Mortality was verified from the cause of death dataset from Taiwan’s Ministry of Health and Welfare [27], per the method in our previous research [28,29].

### 2.4. Statistics

Continuous variables were expressed as their median and interquartile range (IQR) and were evaluated using two-tailed Student’s *t*-tests. Categorical variables were expressed as their frequencies (%) and were analyzed using chi-square tests. Multivariate analysis and the Cox regression model for survival analysis were performed. The strength of associations is presented with hazard ratios (HRs) and 95% confidence intervals (CIs). The survival curves were constructed through Kaplan–Meier analysis. All *p* values were two-sided, and significance was indicated if *p* < 0.05. All analyses were performed using SAS version 9.4 (SAS Institute Inc., Cary, NC, USA).

## 3. Results

### 3.1. Clinical and Demographic Characteristics of Patients

**Figure 1:**
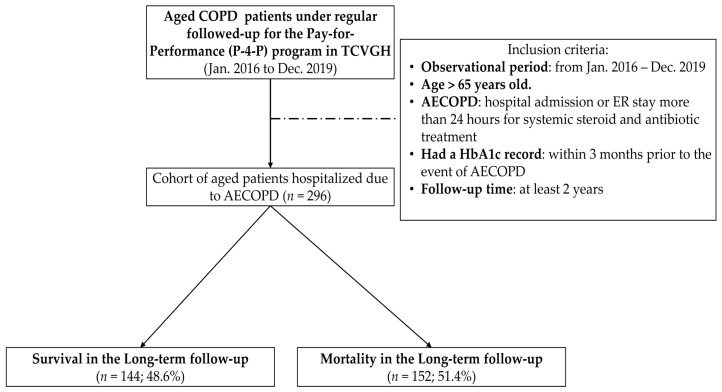
Enrollment and follow-up of the study participants. AECOPD: Acute exacerbation of COPD; COPD: chronic obstructive pulmonary disease; HbA1c: hemoglobin A1c. TCVGH: Taichung Veterans General Hospital; P-4-P: Pay-for-performance.

From January 2016 to December 2017, a total of 296 aged patients with COPD who had been regularly followed up in the P-4-P program of COPD were enrolled for analysis (Figure 1). The demographic characteristics, HbA1c level, comorbidities, medications, and clinical outcomes of patients are presented in Table 1. The median follow-up time for this cohort was 3 years (interquartile range (IQR), 2.6–3.5), which allowed us to track long-term survival. The median age was 80 years old (IQR, 70.0–87.0); for comorbidity status, the study cohort showed 56.4% of patients were comorbid with DM, 16.6% of patients were comorbid with CAD, and 15.9% of patients with cerebrovascular disease (CVA). The short-term outcomes of this cohort were: 23.3% of patients admitted to intensive care unit (ICU); 35.1% of patients experienced respiratory failure requiring mechanical ventilator (MV) support; and eventually, 13.2% of patients died on that episode of hospitalization. Regarding the long-term survival, the overall mortality at 3 years of median follow-up was 51.4% (Table 1).

### 3.2. Differences between Survival and Mortality Groups

The characteristics of the survival and mortality groups were compared (Table 2). Significant differences were only observed in the value of body mass index (BMI), statins prescription, history of ICU admission, and respiratory failure requiring ventilator support between the survival and mortality groups (all *p* < 0.05). For patients in the survival group, these populations were higher in BMI (*p* = 0.003), more with statin prescription (*p* < 0.001), less with ICU admission (*p* < 0.001), and less progression to respiratory failure (*p* < 0.001).

### 3.3. Factors Associated with Long-Term Mortality for Aged Patients with COPD Hospitalized due to AECOPD

**Figure 2:**
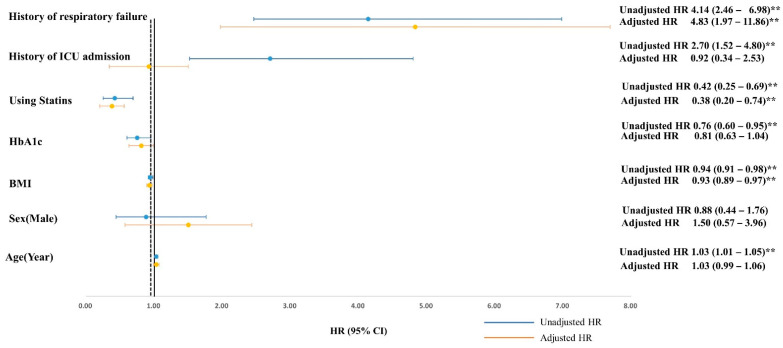
Cox regression model for survival analysis. Multivariate analysis adjusted with BMI, using statin and respiratory failure. ** *p* < 0.01. ICU: intensive care unit.

Table 3 and Figure 2 presents the factors associated with long-term outcome in the Cox regression analysis. Univariate analysis revealed six factors associated with long-term mortality: age (HR, 1.03; 95% CI, 1.01–1.05; *p* < 0.01), BMI (HR, 0.94; 95% CI, 0.91–0.98; *p* < 0.01), HbA1c (HR, 0.76; 95% CI, 0.60–0.95; *p* <0.01), statin prescription (HR, 0.42; 95% CI, 0.25–0.69; *p* < 0.01), ICU admission (HR, 2.70; 95% CI, 1.52–4.80, *p* < 0.001), and progression to respiratory failure (HR, 4.14; 95% CI, 2.46–6.98; *p* < 0.001). By using the multivariate model, we found that that BMI (HR, 0.93; 95% CI, 0.89–0.97, *p* = 0.001), statin prescription (HR, 0.38; 95% CI, 0.20–0.74; *p* =0.004), and progression to respiratory failure (HR, 4.83; 95% CI, 1.97–11.86; *p* = 0.0006) were strong predictive factors associated with long-term mortality of aged patients with COPD after being discharged from hospital (Table 3 and Figure 2).

### 3.4. Analysis of the Effect of Statin Prescription on Overall Mortality of Aged COPD Patients

**Figure 3:**
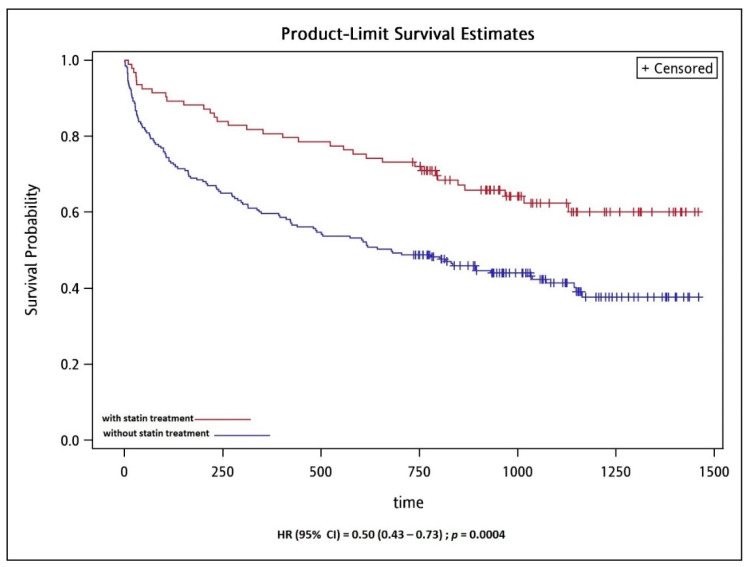
Kaplan-Meier survival curve of overall mortality in aged COPD patients according to use of statins.

The prescription of statins was a strong predictor associated with long-term mortality (adjusted HR: 0.38; 95% CI, 0.20–0.74; *p* = 0.004) (Figure 2); therefore, we conducted the subgroup analysis according to statins prescription or not to create the Kaplan–Meier survival curves and perform the log-rank test on the survival and mortality groups. The Kaplan–Meier analysis indicated patients with statin prescription were significantly associated with lower overall mortality after adjusting factors of age, gender, HbA1c, and BMI (HR: 0.5; 95% CI, 0.34–0.73, *p* = 0.0004) (Figure 3).

### 3.5. Analysis of the Effect of Different Statins on Overall Mortality of Aged COPD Patients

We conducted the subgroup analysis according to the prescriptions of different statins and the associated with long-term outcome in the Cox regression analysis (Table 4). Atorvastatin (52%) was the most common prescription of statin in this cohort. The Univariate analysis revealed only rosuvastatin 10 mg associated with long-term mortality (HR, 0.44; 95% CI, 0.20–0.97; *p* < 0.05).

## 4. Discussion

This study yielded three major findings. First, owing to the regular check-up of comorbidities to compliance the P-4-P program of COPD, we found a high prevalence of cardiovascular and metabolic comorbidities in this aged and hospitalized cohort of COPD; as 56.4% of patients were comorbid with DM, 16.6% comorbid with CAD, and 15.9% comorbid with CVA. Second, we revealed the high impact of AECOPD on short-term health outcomes of this aged cohort, where 13.2% of patients eventually died on that episode of hospitalization, 23.3% of patients were admitted to ICU, and 35.1% of patients experienced respiratory failure requiring ventilator support. The findings from the P-4-P cohort revealed the high healthy impact of AECOPD in short-term outcomes. Third, we found that higher BMI, statin prescription, and no respiratory failure were associated with better long-term mortality on multivariable analysis. In addition, aged patients with COPD prescribed with a statin were associated with better long-term survival by the Kaplan–Meier analysis. Prescribed with Rosuvastatin 10 mg was significantly associated with better long-term outcomes. For aged patients with COPD, the systemic approach for cardiovascular comorbidity and metabolic syndrome for all patients enrolled in the P-4-P program is critical for the prescription of statins to improve long-term outcomes, especially for aged COPD patients who experienced an episode of AECOPD required hospitalization.

The prevalence of cardiovascular diseases in patients with COPD varies between 14% and 33% [30], and our cohort falls within this range. The prevalence of DM in patients with COPD varies from 9% to 30% depending on ethnicity and experimental design [31,32,33,34]; however, few studies have addressed DM prevalence in aged patients with COPD. We found DM prevalence was 56.4% in this cohort is higher than in previous studies [31,32,33,34]. This discrepancy may be due to several factors, including the cohort’s older median age (80; IQR: 70–87), increased severity of COPD (65.2% was Group C, and 34.8% was Group D) according to the GOLD classification [3], and requirement for the intensive followed-up due to the regulation of P-4-P program according to the guidance from National Health Insurance Program. In addition, one publication reported that patients who had AECOPD had a higher DM prevalence (26%) than the general population. Therefore, one important reason for the high prevalence of DM in this cohort was all patients enrolled in this cohort experienced severe AECOPD.

Factors associated with hospitalization [12,13,14,15] and long-term mortality in COPD [16,17] have been widely investigated. In our cohort, the hospital, 1-year, and overall mortalities at a median 3-year of follow-up were 13.1%, 33.8%, and 51.4%, respectively. Regarding factors associated with long-term mortality, we reported the same predictors, such as progression to respiratory failure and ICU admission, as those in studies in Taiwan [14] and Spain [17]. In addition, we discovered that older adults prescribed statins with higher BMI had more promising long-term outcomes. Comorbidity with DM, CAD, CVA was not associated with short-term or long-term mortality, unlike that in other studies [20,35,36]. This result of ours is corroborated by a large prospective registry study of 5334 enrolled patients in China [37]. Patients comorbid with DM tended to incur higher direct medical costs and stay in the hospital longer; however, the in-hospital mortality of patients with COPD comorbid with DM did not increase in the Chinese cohort [37]. Regarding the role of BMI and long-term mortality in COPD, a Chinese study reported that being underweight is a risk factor for people over 75 years old and being overweight reduces all-cause mortality in older adult patients with COPD. A clinical trial conducted in the United States in 2020 indicated that patients with a BMI of more than 30 were associated with reduced mortality of 1.5 years following COPD [37].

Previous studies have shown that statin is associated with reduced mortality in patients with COPD [38,39,40,41]. The most recent study using real-world cohort conducted in the UK showed that patients with statins treatment were associated with a lower risk of death from any cause, HR = 0.63; 95% CI 0.57 to 0.70, after adjustment for all covariates, although the study results showed that statin use was not associated with a lower risk of COPD exacerbation [42]. In this cohort, the first statin prescription was simvastatin (84%), followed by atorvastatin for 14% [42]. Another recent study conducted in Taiwan, using the Taiwan National health insurance database, also showed a significantly lower risk of all-cause mortality for statin-user (HR = 0.76;95% CI = 0.67–0.87, *p* < 0.001) [43]. In this cohort, atorvastatin (44%) was the most commonly used statin, however, only lovastatin (HR = 0.36, 95% CI = 0.14–0.97) and rosuvastatin (HR = 0.51, 95% CI = 0.31–0.82) had a significantly lower risk of mortality [43]. In our subgroup analysis, rosuvastatin (HR = 0.44, 95% CI = 0.20–0.97) was the only statin associated with lower long-term mortality, which revealed that the statins seem to have a differing level of protection and efficacy. The possible mechanism of mortality benefit from using rosuvastatin was suggested through the decreasing of systemic inflammation according to previous landmark studies of the METEOR trial [44] and the JUPITER trial [45]. Our data indicated that a high BMI (median 25.1; IQR 21.2–27.9) and statin prescription, especially rosuvastatin were correlated with higher survival in long-term follow-up after hospitalization (Figure 2 and Figure 3, and Table 4). Therefore, we surmised that higher BMI and satin prescription are good predictors of the long-term survival of aged patients who have COPD.

This study has several limitations. First, each patient may have constituted a heterogeneous sample because of the retrospective design. Second, the sample of this study was enrolled from a single medical center rather than from many medical centers, which limited the generalizability of our findings. Third, biomarkers associated with inflammatory statuses, such as c-reactive protein (CRP) and interleukin (IL)-6, were not routinely checked in the P-4-P program; therefore, we cannot provide the trends of biomarkers before and after AECOPD. In addition, the correlation between statins prescription and biomarkers level was also limited in the current study. Nonetheless, all patients attended a referral medical center and received standardized medical care for COPD, which reduces the confounding effects of mortality in this study. One important strength of the study may support the value of findings from this cohort. The COPD Pay-for-Performance program as part of the National Health Insurance Program was enacted in Taiwan; each patient enrolled in the program ought to be followed up regularly by case managers in relation to inhaler usage and medications, pneumococcus vaccinations, and pulmonary rehabilitation and comorbidity survey. Thus, contamination from differing care plans or treatments is less likely in this cohort. Moreover, the study used real-world data rather than claims data, which means that the results are more reflective of the patient’s status and more useful to clinicians.

## 5. Conclusions

Long-term mortality is high for aged and hospitalized patients with COPD who experienced AECOPD. The prevalence of DM, CAD, and CVA of patients were 54%, 16.6%, and 15.9%, respectively, in our study cohort from the P-4-P program. In addition, we found higher BMI, with statin prescription and without respiratory failure, were significantly associated with better long-term survival of aged patients with COPD after discharging from the hospital. Rosuvastatin 10 mg was associated with lower mortality. For aged and hospitalized patients with COPD, statins prescription may associate with better long-term outcomes according to this real-world cohort from the P-4-P program.

## Figures and Tables

**Table 1:** Demographic characteristics, baseline glucose status, comorbidities, and outcomes of geriatrics who experienced a severe acute exacerbation of COPD required hospitalization (*n* = 296).

Variables	Median	IQR
Age (year)	80.0	70.0–87.0
Gender-Male (*n*, %)	259	87.5%
Body mass index (kg/m^2^)	23.7	20.6–27.2
HbA1c	6.5	5.9–7.5
Pulmonary function test		
FEV1 (%)	68.0	53.0–87.0
FVC (%)	86.0	72.0–108.0
FEV1/FVC (%)	63.0	53.0–71.0
GOLD classification		
Category C (*n*, %)	193	65.2%
Category D (*n*, %)	103	34.8%
Comorbidity Status (Deyo Score ≥ 1)	190	64.2%
Coronary artery disease (CAD) (*n*, %)	49	16.6%
Cerebrovascular disease (CVA) (*n*, %)	47	15.9%
With DM history (*n*, %)	167	56.4%
Using Insulin or OHA (*n*, %)	99	33.5%
Using Statins (*n*, %)	93	31.4%
Outcomes		
ICU admission (*n*, %)	69	23.3%
Respiratory failure (*n*, %)	104	35.1%
Hospital mortality (*n*, %)	39	13.2%
1-year mortality (*n*, %)	100	33.8%
Overall mortality (*n*, %)	152	51.4%
Follow-up years	3.0	2.6–3.5

**Table 2:** Factors associated with overall mortality with median 3-year follow-up time of aged COPD patients required hospitalization (*n* = 296).

Variables	Total (*n* = 296)	Mortality (*n* = 152)	Survival (*n* = 144)	*p* Value
Median (IQR); (*n*, %)				
Age (year)	80.0 (70.0–87.0)	81.0 (74.0–88.0)	78.0 (68.0–86.0)	0.051
Sex-Male ^c^	259 (87.5%)	134 (88.2%)	125 (86.8%)	0.725
BMI (kg/m^2^)	23.7 (20.6–27.2)	22.0 (20.0–26.1)	25.1 (21.2–27.9)	0.003 **
HbA1c (%)	6.5 (5.9–7.5)	6.4 (5.9–7.3)	6.9 (6.0–7.7)	0.3949
Pulmonary function test				
FEV1 (%)	68.0 (53.0–87.0)	70.5 (55.0–87.0)	65.0 (53.0–87.0)	0.755
FVC (%)	86.0 (72.0–108.0)	86.5 (77.0–108.0)	85.0 (67.0–102.0)	0.533
FEV1/FVC (%)	63.0 (53.0–71.0)	60.5 (52.0–69.0)	64.0 (55.0–72.0)	0.189
GOLD classification				
Category C (*n*, %)	193 (65.2%)	99 (65.1%)	94 (65.3%)	0.979
Category D (*n*, %)	103 (34.8%)	53 (34.9%)	50 (34.7%)	
Comorbidity Status				
Deyo Score ≥ 1	190 (64.2%)	92 (60.5.0%)	98 (68.1%)	0.178
CAD (*n*, %)	49 (16.6%)	28 (18.4%)	21 (14.6%)	0.375
CVA (*n*, %)	47 (15.9%)	21 (13.8%)	26 (18.1%)	0.319
With DM history ^c^	167 (56.4%)	82 (54.0%)	85 (59.0%)	0.379
Using Insulin or OHA ^c^	99 (33.5%)	51 (33.6%)	48 (33.3%)	0.968
Using Statins ^c^	93 (31.4%)	34 (22.4%)	59 (41.0%)	<0.001 **
History of ICU admission ^c^	69 (23.3%)	48 (31.6%)	21 (14.6%)	0.001 **
History of respiratory failure ^c^	104 (35.1%)	76 (50.0%)	28 (19.4%)	<0.001 **

^c^ Chi-square test. Mann–Whitney U test. ** *p* < 0.01. BMI: Body mass index; PFT: pulmonary function test; FEV1: forced expiratory volume in one second; FVC: forced vital capacity; GOLD: Global Initiative for Chronic Obstructive Lung Disease; DM: diabetes mellitus; ICU: intensive care unit.

**Table 3:** Cox analysis of factors associated with long-term outcome in aged COPD patients required hospitalization.

Variables	Univariate Analysis HR (95% CI)	Multivariate Analysis HR (95% CI)
Age (year)	1.03 (1.01–1.05) **	1.03 (0.99–1.06)
Sex-Male	0.88 (0.44–1.76)	1.50 (0.57–3.96)
BMI	0.94 (0.91–0.98) **	0.93 (0.89–0.97) **
HbA1c	0.76 (0.60–0.95) **	0.81 (0.63–1.04)
Deyo Score ≥ 1	0.72 (0.45–1.16)	
With DM history	0.81 (0.51–1.29)	
CAD	1.32 (0.71–2.45)	
CVA	0.73 (0.39–1.36)	
Using Insulin or OHA	1.01 (0.62–1.64)	
Using Statins	0.42 (0.25–0.69) **	0.38 (0.20–0.74) **
History of ICU admission	2.70 (1.52–4.80) **	0.92 (0.34–2.53)
History of respiratory failure	4.14 (2.46–6.98) **	4.83 (1.97–11.86) **

Cox regression. ** *p* < 0.01. Multivariate-2: adjusted BMI, using statins and history of respiratory failure. CI: confidence interval; DM: diabetes mellitus; HR: hazard ratio; ICU: intensive care unit.

**Table 4:** The prescription patterns and the associated with long-term outcome in the Cox regression analysis.

Statin Type and Dose (*n* = 93)	*n* (%)	Univariate Analysis HR (95% CI)
Rosuvastatin 10 mg	30 (32.3%)	0.44 (0.20–0.97) *
Pravastatin 40 mg	15 (16.1%)	0.33 (0.10–1.05)
Atorvastatin 10 mg	13 (14.0%)	0.80 (0.26–2.45)
Atorvastatin 20 mg	34 (36.5%)	0.63 (0.31–1.30)
Atorvastatin 40 mg	1 (1.1%)	n/a
Atorvastatin all dose	48 (52%)	0.70 (0.37–1.29)

Cox regression. * *p* < 0.05. Note. The univariate analysis in Atorvastatin 40 mg is limited due to only 1 prescription.

## Data Availability

The data presented in this study are available on request from the corresponding author. The data are not publicly available due to the regulation of the Institutional Review Board of Taichung Veterans General Hospital in Taiwan.
